# The Efficacy of Optimism: Benefit Finding in the Treatment of Diabetes in Iranian Patients

**DOI:** 10.1155/2014/371296

**Published:** 2014-03-18

**Authors:** Hossein Karimi Moonaghi, Hossein Namdar Areshtanab, Leila Jouybari

**Affiliations:** ^1^Department of Medical-Surgical Nursing, Faculty of Nursing and Midwifery, Mashhad University of Medical Sciences, Mashhad 9137913199, Iran; ^2^Department of Postgraduate, Faculty of Nursing and Midwifery, Mashhad University of Medical Sciences, Mashhad 9137913199, Iran; ^3^Faculty of Nursing and Midwifery, Golestan University of Medical Sciences, Gorgan 49155568, Iran

## Abstract

The incidence of diabetes mellitus is on the rise around the world. Middle Eastern countries will be facing a vast increase in the number of diabetes mellitus cases by 2030. Diagnosis of a chronic disease such as diabetes mellitus can be a shocking and life-altering event. Conversely, a diagnosis of a chronic illness can also offer the patient opportunities to change unhealthy behaviors such as poor diet, smoking, and lack of exercise, making them healthier than before their diagnosis. This is referred to as “benefit finding”. This study reveals the many benefit findings of Iranian patients who have been diagnosed with diabetes mellitus and illustrates how benefit finding can be an integral part of long-term patient care.

## 1. Introduction

Diabetes mellitus is one of the most challenging and burdensome chronic diseases of the 21st century, and it is a growing threat to the world's public health [1]. Diabetes mellitus currently affects about 285 million adults worldwide and this figure is expected to rise to over 400 million adults by 2030 [2]. Type 2 diabetes mellitus is responsible for over 90% of all cases of diabetes [3]. Most new patients with diabetes are from developing countries and it seems that the Middle East is among the regions that will have the largest increase in the prevalence of diabetes by 2030. According to the research results, the prevalence of diabetes is about 8.7% in Iranians aged 25–64 years (9.2% in women and 7.5% in men). More than 1% of the Iranian urban population older than 20 years develops diabetes mellitus each year [4]. Diabetes mellitus can lead to serious complications and premature death [5]. Consequences of chronic diabetes include eye disease, circulatory problems and kidney failure, decreased quality of life, and increased use of health services [6]. The diagnosis of a life-threatening illness, such as diabetes, can be an extremely stressful and traumatic experience [7]. Studies have showed that living with diabetes has a negative impact on many aspects of daily life and the quality of life for people with diabetes [8].

Although negative outcomes of having an acute or chronic illness have received the most attention, studies can be found elucidating possible positive effects of serious illness [9]. However, some emerging evidence has shown that experience with life-threatening adversity can be an accelerator for personal growth and transformation. For example, research has found that patients may change the way they view themselves, their priorities, and their interactions with others in response to a health-related trauma [10].

The manner in which patients perceive positive of negative implications of their illness can influence various psychological consequences [11]. Posttraumatic growth could be one of the cognitive strategies used to deflect the negative effects of illness. When diagnosed, individuals may change their internal standards of what constitutes good health or other aspects of quality of life (recalibration), adjust their values and priorities (reprioritization), or redefine what they think is important (reconceptualization) to maintain an acceptable quality of life in the face of declining health [12]. Benefit finding has been associated with better psychosocial well-being and lower affective distress in numerous chronic illness populations. A review of the literature showed that the benefit findings are typically investigated in diseases such as cancers, spinal cord injuries, multiple sclerosis, and rheumatoid arthritis with only a few studies conducted among myocardial infarction and HIV/AIDS patients [13, 14]. On the other hand, several studies have been conducted on type 2 diabetes patients [15]. Available literature is based on studies conducted in western societies that differ in terms of cultural and social backgrounds from the Iranian society. Moreover, the studies have shown that sociocultural context could influence illness and outcome of the process of adaptation to disease [16, 17].

An understanding of benefit finding is significant in the proper provision of health care, such as nursing, as it appears that benefit finding aids in dealing effectively with illness and plays a prominent role in the cognitive process that facilitates adjustments to adversity [18]. The aim of this study is to explore positive change experiences of patients with diabetes in the Iranian population.

## 2. Materials and Methods

A purposive sample of fifteen patients was recruited. Patients were selected on the basis of the following inclusion criteria: a confirmed diagnosis of type 2 diabetes mellitus for at least two years; awareness of their diagnosis; cognitive and physical ability to participate; and a willingness to participate. Data were collected via in-depth, semistructured, and face to face interviews conducted between September 2011 and August 2012. All interviews were private and conducted at the participant's discretion with regard to place and time by the Diabetes Association of Iran, Tabriz branch. The interviews were conducted in Persian by corresponding author. Each participant was interviewed only once. The analysis was conducted in Persian and, for the purposes of this paper, translated into English. The interviewer asked the participants about their experiences of living with diabetes. The interviews were tape-recorded and transcribed verbatim. The first set of interviews lasted 60–80 minutes and the next set of interviews lasted 40–50 minutes (mean time of each interview is 45 minutes). Data collection continued until data saturation was achieved. That is, data collection had gone on up to when no new code emerged from analysis of the data. The analysis of the interview texts began once the first interview had been conducted and transcribed. Data were analyzed using qualitative content analysis techniques inspired by Graneheim and Lundman [19]. The analysis began by reading through each interview several times to obtain a sense of the whole of the participants' experiences. Then, the meaning units, which were words, sentences, or paragraphs that depicted important aspects of participants' experiences, were highlighted. These meaning units were then condensed to shorten the text but retain the content. In the next stage, these condensed meaning units, or codes, were abstracted from each interview transcript. Finally, using comparison, reflection, and interpretation, these codes were grouped into categories and subcategories by the first three authors. Criteria for enhancing the rigor of qualitative studies have been proposed by Lincoln and Guba and include credibility, transferability, dependability, and confirmability [20]. In the research process, researchers allocated sufficient time for data collection and retained close communication with study participants. The interviews were returned to the participants for verification of the accuracy of the results and to validate the congruity of the interpretive findings with their experiences. This ensured that researchers were correctly representing the ideas of the patients.

The data were coded and categorized independently by the authors and emergent themes were compared. Opinions of experts and also three Ph.D. candidates of nursing in data analysis as peer reviewers were used and cases were discussed over a two-week period. Regarding rigor, the research team discussed and interpreted the findings until an agreement was reached, wherein one author collected and analyzed the data (Hossein Namdar Areshtanab) and two researchers (Hossein Karimi Moonaghi, Leila Jouybari), who had expertise and were interested in conducting qualitative research, discussed, checked, and verified the meanings which were being emerged. These reviews enhanced the credibility of the data. To increase the dependability, one of the researchers collected and analyzed the data and other researchers checked and verified the results. Based on data collected from participants and memos taken after reading each of the interviews, participants were selected by considering maximum variation in terms of age, gender, previous experience with illness, family economic status, educational level, job background, and requirement of daily injectable insulin to provide transferability. Following the approval by the Ethics Committee of the Mashhad University of Medical Sciences, informed consents were obtained from all participants who met inclusion criteria for the study. All participants were assured that their personal data would remain confidential. Time and location were chosen by the agreement of the participants. Examples of interview questions are as follows.

1. Can you tell us about your experiences of living with diabetes mellitus?
2. Can you describe some things you did or experienced that were helpful in the management of your disease?
3. What positive effects/changes do you feel may have occurred in your life due to diabetes mellitus?

## 3. Finding

Of the participants, 9 (60%) were males and the average age was 47.8 ± 12.0 years and 13 (86.7%) were married. Six (40%) had a lower educational background (illiterate to high school) and 6 (40%) had higher education backgrounds (college graduates). Females had a lower educational background than males. Most females were unemployed while most males were employed and three of them were retired. Participants averaged 8.7 years living with a diagnosis of diabetes and 8 (53.3%) participants required daily insulin injection. Most of the participants (60%) were not members of the Diabetes Association (Table 1).

During the data analysis, three main themes emerged as the positive experiences of Iranian patients with diabetes: lifestyle modification; promotion of interpersonal interactions; and appreciating health (Table 2).

### 3.1. Lifestyle Modification

The participants considered their diseases as an agent in changing unhealthy lifestyle behaviors. This refers to positive changes in knowledge, behavior, self, and spiritual dimensions of participants. The category has four subcategories: promotion of knowledge; modification of behaviors; promotion of self; and promotion of spiritual aspects.

#### 3.1.1. Promotion of Knowledge

Diabetes was seen as an agent in increasing personal knowledge and information about this disease and the proper functioning of organs of the body. The participants had not been aware of the disease but they had relative knowledge.

**I thought that eating too much sugar causes diabetes or if my doctor says that I need to start using insulin, it means I failed to take care of my diabetes properly. However, now I know that type 2 diabetes mellitus is cause by genetics and lifestyle factors such as stress and lack of mobility… Or over time, the body gradually produces less and less of its own insulin and eventually oral medications may not be enough to keep blood glucose levels normal. Using insulin to get blood glucose levels to a healthy level is a good thing, not a bad one. (Man, aged 54)**

#### 3.1.2. Modification of Behaviors

This subcategory presents statements of participants that diabetes gave them an opportunity to change their unhealthy habits, which they perceive as ruining their health but did not previously have enough willpower or incentive to stop. Also it provided an opportunity for patients who were sensitive to their health to adjust their life goals and expectations.

**I already had a low physical activity level and I ate fatty and fried foods. Now I try to walk every day for at least one half hour and I eat more low-fat and low cholesterol foods such as vegetables and fruits. (Woman, aged 50)**

**I was already overweight and smoking. I was not able to reduce my weight or stop smoking. I realized after diabetes that if I wanted to continue living, I should quit smoking and lose weight and I got it. (Man, aged 45)**

**Following my illness, I regularly check my blood glucose, cholesterol, triglycerides and blood pressure. (Man, aged 52)**

**Before my illness, I wanted to work after retirement. Now I want to retire earlier so that I may buy a garden where I can plant trees and get relaxation. (Man, aged 57)**

#### 3.1.3. Promotion of Self

Diabetes mellitus was seen as an agent in modifying coping styles, identification with successful people, and an increase in empathy (feeling more compassionate to others).

**Now, I prepare to participate in scientific studies about diabetes mellitus in order to progress in the treatment of it, while previously I had no such intention (meaning based coping). (Man, aged 43)**

**For forty-eight years my aunt has had type 2 diabetes mellitus and she lives comfortably with it. I always use her as a model on life with diabetes and I use her guidance in diabetes control.” (Identification). (Woman, aged 61)**

**Before becoming ill, some of behaviors in diabetes such as stated discomfort of repeated injections of insulin or rigid diet were pointless for me. Now, I'm less judgmental because I realize that diabetics may have many reasons for their behaviors (Empathy). (Man, aged 50)**

#### 3.1.4. Promotion of Spirituality

Diabetes mellitus was seen as an agent in devoting more attention to spiritual issues. The participants were paying more attention to their spirituality following their illnesses. They believed that a closer relationship with God and greater spirituality could help them better tolerate the illness.

**After I prayed and read the Koran, I feel inner peace and I better cope with the problems of life and diabetes. (Man, aged 49)**

**I begin my life with God's name every day. God is my real helper. (Woman, aged 41)**

### 3.2. Promotion of Interpersonal Interactions

The participants considered this disease as the agent in changing interpersonal relations. It refers to positive changes in relationships between family members, participation in group activities, and empathy with other patients with diabetes.

**Following my illness and over time, my wife has learned to comfort me and in the family nutrition programs, she is also considering my nutritional circumstances. The children understand my situation now and they support me. (Man, aged 62)**

**Following my illness, I am participating in educational classes of the Diabetes Association where I share my experiences with other patients. I have become familiar with the newest treatments for diabetes mellitus. I am participating in the group exercises with other patients. (Man, aged 38)**

**Before having diabetes mellitus, when I saw a patient with diabetes, I told myself that this is a very minor illness, but now when I see someone with diabetes mellitus I am very sad and upset. I tell myself it isn't a wound that you can open and close easily, nor is it a dislocation of a joint. It is really painful and hard to tolerate. (Man, aged 55)**

### 3.3. Appreciating Health

Participants believed that the illness has helped them appreciate things that previously were not important to them. It refers to positive changes in using opportunities in life and realizing the value of health.

** Following my illness, I realized that life is short and every moment of life is precious. (Woman, aged 65)**

**After this illness, I understood that good health is a great blessing and it is the most valuable thing in my life. (Man, aged 39)**

The study was affected by duration of illness, family support, severity of illness, educational level, economic status, beliefs, and underlying diseases of the participants.

**I know nothing will happen to me except God willing. Maybe my illness is a kind of test from God in my life. When I think deeply, I find out that this illness has got some benefits for me. (Woman, aged 62)**

The research findings are shown in Table 2.

## 4. Discussion and Conclusion

Until now, there have been no studies conducted in Iran on posttraumatic growth following type 2 diabetes mellitus. This study shows that the participants have realized positive changes following type 2 diabetes mellitus depending on what meaning they ascribe to it. Studies have shown that how the individual cognitively processes information plays an essential role in coping with illness [21].

In this study, one of the positive changes after a diagnosis of type 2 diabetes mellitus was promotion of lifestyle. It was evidenced in knowledge, behavior, self-, and spiritual dimensions of participants. The present study found promotion of spiritual aspects as the most significant dimension of benefit finding. Considering the dominant religious culture in Iran and special role of religion and spirituality in facing stressful events, predominance of the spiritual promotion subtheme is quite justified. Thus, taking into account existing studies, it seems that promotion of lifestyle is one of the positive outcomes to chronic illness [15, 22–25].

In this study, the illness may not only influence the patients to change their lifestyles, but also affect the interpersonal interactions in relation to positive changes in relationships between family members, participation in group activities, and empathy with other patients with diabetes. Thus, taking into account existing studies, it seems that positive changes in relationships are one of the positive outcomes to chronic illness [15, 18, 26–28].

Another positive change in the aftermath of the illness was appreciation of life. Thus, taking into account existing studies, it seems that appreciation of health is one of the positive outcomes to chronic illness [18, 23, 29]. In this study, length of time with the disease, high family support, low severity of illness, high educational level, high economic status, religious beliefs, and no underlying disease had positive impacts on posttraumatic growth in diabetes. Thus, taking into account existing studies, it seems that these factors have a positive effect on diabetes mellitus [30].

It can be concluded that, despite the difficulties of having diabetes, it may also have positive effects for those diagnosed. Consideration of the effects of the disease on patients' life experiences and understanding of their thoughts about the disease as well as the disease's impact on adaptation to disease are important elements in nursing care and education.

## 5. Limitations and Recommendations

In this study, the sample size is small and was carried out in one national context. Also, the findings reflect only the view of patients regarding positive effects of type 2 diabetes mellitus. Thus, as with other such qualitative studies, caution is needed in generalizing the findings to either Iran as a country or other countries around the world. Also, future study requires input from both health care providers and families. We believe that it is necessary to conduct a population-based survey to confirm these findings.

## Figures and Tables

**Table 1:** Sociodemographic and clinical characteristics of participants.

Gender	Mean (SD) or number (%)
Male	9 (60%)
Female	6 (40%)
Age	47.86 ± 12
Marital status, number (%)	
Married	13 (86.6%)
Single	1 (6.7%)
Widowed	1 (6.7%)
Employment, number (%)	
Unemployed	3 (20%)
Employed	7 (46.7%)
Retired	5 (33.3%)
Education level, number (%)	
Illiterate to under diploma	6 (40%)
diploma	3 (20%)
Graduate	6 (40%)
Type of treatment, number (%)	
Oral medication	9 (60%)
Insulin	8 (53/3%)
Both medications	6 (40%)
Insurance, number (%)	
Covered	15 (100%)
Not covered	0
Income	
Low	5 (40%)
Medium	6 (33.3%)
High	4 (26.7%)
Duration of diabetes, mean (S.D)	8.7 ± 5.07 (1–25)
Family history	
Yes	9 (60%)
No	6 (40%)
Member of the Diabetes Association	
Yes	9 (60%)
No	6 (40%)

**Table 2:** 

Them	Subtheme/frequency and percentage
lifestyle modification	Promotion of knowledge (*n* = 13, 86.6%)
	Modification of behaviors (*n* = 12, 80%)
	Promotion of self (*n* = 10, 66.6%)
	Promotion of spiritual aspects (*n* = 14, 93.3%)

Promotion of interpersonal interactions	Promotion relationships between family members (*n* = 11, 73.3%)
	Participation in group activities (*n* = 10, 66.6%)
	Empathy with other patients with diabetes (*n* = 10, 66.6%)

Appreciating life	Taking advantage of opportunities in life (*n* = 12, 80%)
	Worthy of health (*n* = 13, 86.6%)
